# Epigenetic Regulation of Antibody Responses by the Histone H2A Deubiquitinase MYSM1

**DOI:** 10.1038/srep13755

**Published:** 2015-09-08

**Authors:** Xiao-Xia Jiang, YuChia Chou, Lindsey Jones, Tao Wang, Suzi Sanchez, Xue F Huang, Lei Zhang, Changyong Wang, Si-Yi Chen

**Affiliations:** 1 Department of Molecular Microbiology and Immunology, Norris Comprehensive Cancer Center Keck School of Medicine, University of Southern California, Los Angeles, California, 90033, USA; 2 Department of Advanced Interdisciplinary Studies, Institute of Basic Medical Sciences, Beijing, 100850, China

## Abstract

B cell-mediated antibody response plays critical roles in protective immunity, as well as in the pathogenesis of allergic and autoimmune diseases. Epigenetic histone and DNA modifications regulate gene transcription and immunity; however, so far, little is known about the role of epigenetic regulation in antibody responses. In this study, we found that mice deficient in the histone H2A deubiquitinase MYSM1, despite their severe defect in B cell development, exhibit an enhanced antibody response against both T cell-dependent and independent antigens. We revealed that MYSM1 intrinsically represses plasma cell differentiation and antibody production. Mechanistic studies demonstrated that MYSM1 is a transcriptional activator of Pax5, the repressors of plasma cell differentiation, by facilitating key transcriptional factor recruitment and coordinating histone modifications at the Pax5 loci. Hence, this study uncovers a critical role for MYSM1 in epigenetically repressing plasma cell differentiation and antibody production, in addition to its opposing, active role in B cell development. Importantly, this study further provides a new target and strategy to modulate antibody production and responses with profound therapeutic implications.

Genomic DNA is compacted through its association with histone proteins in an octamer, consisting of two copies of histones H2A, H2B, H3, and H4, to form nucleosomes and chromatin. Histone and DNA modifications determine chromatin structure, while maintaining distinct transcription patterns, and cellular identity and functions^1^,^2^,^3^,^4^. Histones are subject to a variety of post-translational modifications, including methylation, acetylation, phosphorylation, sumoylation, and ubiquitination^1^,^5^. Various enzymes catalyze histone modifications, while an increasing number of enzymes that catalyze the removal of these histone marks have been recently identified^1^,^5^, indicating that epigenetic histone modifications are a reversible and highly dynamic process. Recent studies demonstrate that epigenetic histone and DNA modifications at target transcription factor and cytokine loci are of importance in the process of T lymphocyte lineage differentiation and functions^6^,^7^,^8^,^9^. However, to date, little is known about the epigenetic regulation of B cell differentiation and antibody responses.

Histone H2A is monoubiquitinated at the conserved residue lysine (K) 119 by histone H2A ubiquitinases^10^,^11^,^12^, which represents a non-degradative, epigenetic signal^5^,^13^. Recently, numerous histone H2A deubiquitinases, including MYSM1, USP16/Ubp-M, USP21, USP22, and PR-DUB/BAP1, have been identified^14^,^15^,^16^,^17^,^18^. H2A deubiquitination activity of the Myb-like, SWIRM, and MPN domains-containing protein 1 (MYSM1) is associated with target gene transcription^17^. The JAMM/MPN domain possesses an intrinsic metalloprotease activity that hydrolyzes the isopeptide bonds of ubiquitin chains, while the SANT domain is similar to the DNA-binding domain of Myb-related proteins^19^ and the SWIRM domain frequently exists in the members of the SWI/SNF-family of ATP-dependent chromatin remodeling complexes^20^. In a recent study, we found that MYSM1 is essential for B cell development by derepressing the transcription of EBF1, Pax5, and other B-lymphoid genes^21^. Mechanistic studies revealed that MYSM1 is an epigenetic transcriptional switch that orchestrates histone modifications and transcription factor recruitment to the target EBF1 locus. The mature B cell compartment is composed of follicular (FO), B1, and marginal zone (MZ) B cells^22^,^23^,^24^, which are located in distinct anatomical sites. B1 B cells are found in the pleural and peritoneal cavities, and MZ B cells reside within the splenic white pulp. B1 B cells and MZ B cells act to mediate the initial wave of humoral immunity against invading pathogens by quickly producing low affinity, antigen-specific IgM antibodies in a thymus-independent (TI) fashion. In contrast, FO B cells comprise the majority of B cells found in peripheral lymphoid organs and respond to antigens in a thymus-dependent (TD) manner^22^,^23^,^24^. In this study, we unexpectedly observed that MYSM1-deficient mice had an enhanced antibody response despite the severe defect in B cell development. Mechanistic studies revealed that MYSM1 intrinsically represses plasma cell differentiation and antibody production by activating the transcription of Pax5, the repressors of plasma cell differentiation, in mature B cells. Furthermore, this study provides a new strategy and target to modulate antibody production and responses with profound therapeutic implications.

## Results

### Enhanced primary and recall antibody responses in Mysm1^−/−^ mice despite the severe defect in follicular (FO) B cell development

**Figure 1:**
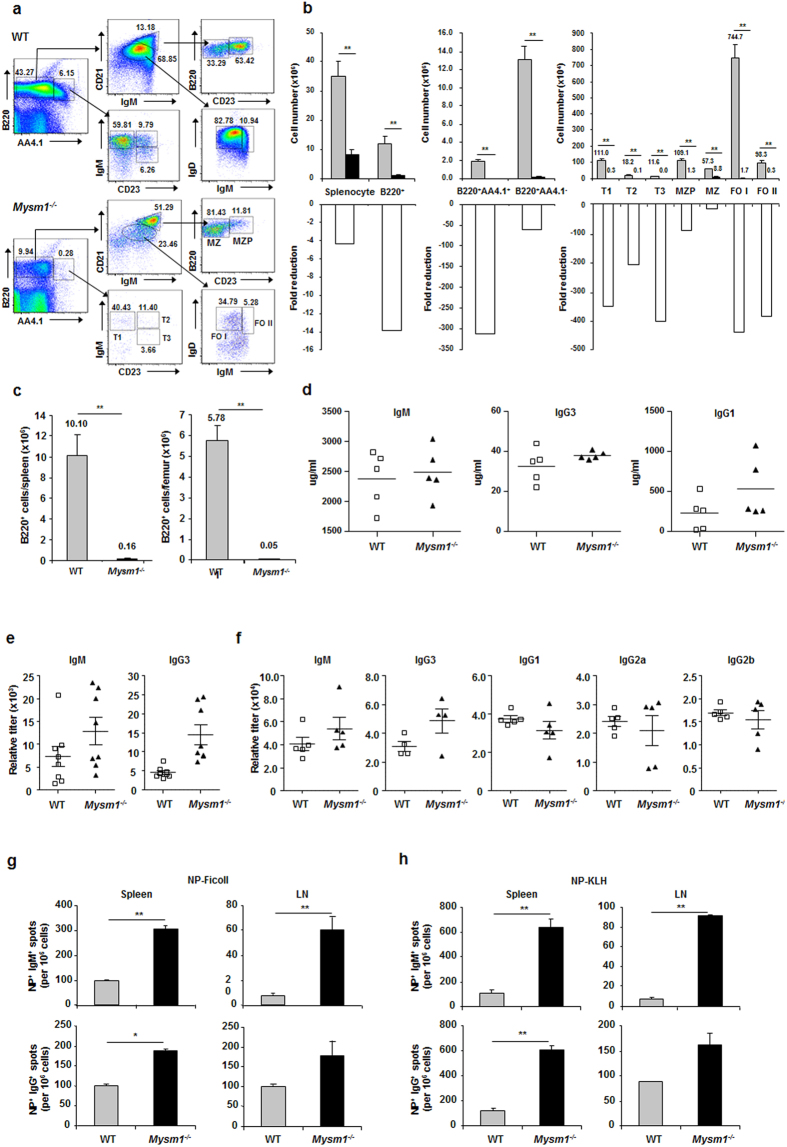
Enhanced primary TI and TD antibody responses in Mysm1^−/−^ mice despite the severe defect in FO B cell development. (**a**) Representative flow cytometry analysis of WT and Mysm1^−/−^ splenocytes stained with indicated antibodies from four independent experiments. Numbers are the percentage of events within the indicated gates. (**b**) Absolute numbers (top) of indicated B cell subsets in spleen of Mysm1^−/−^ mice and WT littermates and fold reduction (bottom) of Mysm1^−/−^ cell numbers compared to WT cell numbers (n = 5–8 per group) from one of three independent experiments. **P < 0.01, WT *vs.* Mysm1^−/−^. (**c**) Graphs show the absolute numbers of B220^+^ B cells in the spleen (left) and bone marrow (right) from WT and Mysm1^−/−^ mice. **P < 0.01, WT *vs.* Mysm1^−/−^. (**d**) Serum from naïve WT and Mysm1^−/−^ mice was analyzed for resting levels of IgM, IgG3, and IgG1 by ELISA. (**e**) Mice were immunized with NP-Ficoll (50 μg) and serum was collected at day 14 for examining NP-specific IgM and IgG3 levels by ELISA with plates coated with NP26-BSA. (**f**) WT and Mysm1^−/−^ mice were immunized with NP-KLH (100 μg) precipitated in Alum. Serum was harvested at day 14 after immunization to quantitate NP-specific IgM, IgG3, IgG1, IgG2a, and IgG2b antibodies by ELISA. (**g**,**h**) ELISPOT analysis of anti-NP IgM (top) and IgG (bottom) production by cells pooled from spleens or lymph nodes of WT and Mysm1^−/−^ mice 14 d after immunization with NP-Ficoll (**g**) or NP-KLH (**h**). **p < 0.01, *p < 0.05, Mysm1^−/−^ *vs.* WT.

In the absence of MYSM1, there is a block in early B cell development with a severe reduction in the frequency and absolute number of both peripheral immature and mature B cells^21^. In order to further define the role of MYSM1 in peripheral B cell subpopulation development, we analyzed splenic subpopulations of B cells in WT and Mysm1^−/−^ mice by flow cytometry. We observed a drastic decrease in the percentages and numbers of immature, transitional B-lineage precursor marker CD93/AA4.1^+^ B cell populations (IgM^+^CD23^−^ (T1), IgM^+^CD23^+^ (T2), and IgM^lo^CD23^+^ (T3)) in the spleens of Mysm1^−/−^ mice relative to WT controls (Fig. 1a,b). Frequencies of both B220^+^CD93/AA4.1^lo^ mature B cell and B220^+^CD93/AA4.1^high^ immature B cell populations, and absolute B220^+^ B cell numbers in the spleen and bone marrow of Mysm1^−/−^ mice were drastically reduced (Fig. 1a–c). We further observed a drastic reduction in both the percent and cell number of CD21^lo^ FO B cells (FO I and FO II) in the spleens of Mysm1^−/−^ mice. However, the percentages of CD21^hi^ MZ B cells were increased in the spleens of Mysm1^−/−^ mice. The absolute cell numbers of both MZP and MZ B cells were reduced at lesser degrees in the Mysm1^−/−^ mice. Thus, these data demonstrate a severe defect in the development of follicular B cells, but a much milder developmental defect in MZ B cells in Mysm1^−/−^ mice.

When examining the basal levels of serum Immunoglobulin (Ig) in naïve Mysm1^−/−^ mice by ELISA, we were surprised to find that the serum concentrations of IgM and IgG isotypes were normal or even higher in Mysm1^−/−^ mice, compared to those in WT controls (Fig. 1d). To investigate the possible role of MYSM1 in antibody responses, Mysm1^−/−^ mice and WT littermates were immunized once with NP-Ficoll (TI antigen), NP-KLH (TD), or KLH control in alum and sera were analyzed by ELISA for NP-specific antibodies. It was found that both TI and TD anti-NP antibody responses, including anti-NP IgM, IgG1, IgG2a, and IgG3, were comparable or enhanced in Mysm1^−/−^ mice (Fig. 1e,f), despite a severe reduction in the peripheral B cell population (Fig. 1a–c). ELISPOT assays were performed to determine if the increase in serum Ig was due to an increase in Ig secreting cells in immunized Mysm1^−/−^ mice. Figure 1g,h show that there was a great increase in the frequencies of NP-specific IgM and IgG secreting cells in the Mysm1^−/−^ mice that were immunized with either TI NP-Ficoll or TD NP-KLH antigens.

**Figure 2:**
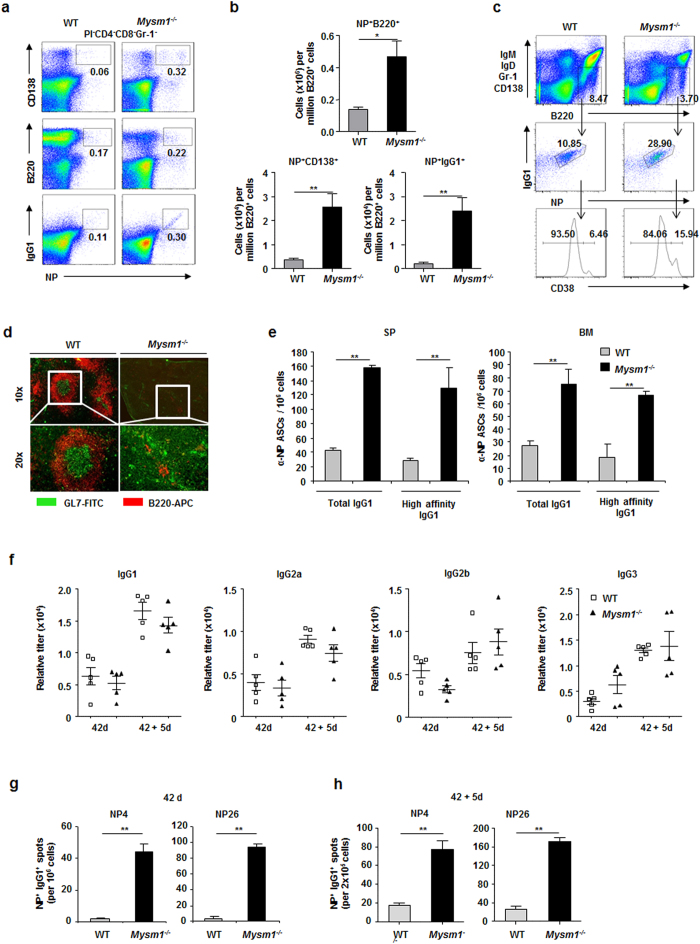
Enhanced recall TD antibody responses in Mysm1^−/−^ mice. (**a**,**b**) Flow cytometry analysis of splenocytes of WT and Mysm1^−/−^ mice 14 days after intraperitoneal immunization with NP-KLH (100 μg) in alum. PI^−^CD4^−^CD8^−^Gr-1^−^ cells were analyzed for NP^+^CD138^+^, NP^+^B220^+^, and NP^+^IgG1^+^ cells (**a**). Numbers indicate the percent in each. Absolute cell numbers of NP^+^B220^+^ B cells, NP^+^CD138^+^ plasma cells, and NP^+^IgG^+^ antibody-producing cells per million B220^+^ cells for each group (n = 5–8 per group) 14 days after primary intraperitoneal immunization with NP-KLH (100 μg) precipitated in alum from one of three independent experiments (**b**). **P < 0.01, WT *vs.* Mysm1^−/−^. (**c**) Flow cytometry analysis of splenocytes 14 days after intraperitoneal immunization with NP-KLH in alum. Isotype-switched B cells (IgM^−^IgD^−^Gr^−^1^−^CD138^−^B220^+^) were analyzed for NP^+^IgG1^+^ status with NP^+^IgG1^+^ cells being subdivided into GC (CD38^−^) and memory (CD38^+^) B cells. Numbers in plots and histograms represent percentage of cells within the gate. (**d**) Frozen spleen sections from WT and Mysm1^−/−^ mice 14 days after immunization with NP-KLH, stained with antibodies to B220 to identify follicles (red) and GL7 for germinal centers (green). Original magnification is x10 (top) and x20 (bottom). (**e**) Frequencies of total (NP26) and high-affinity (NP4) NP-specific-secreting ASCs in spleen and bone marrow examined by ELISOPT assays. Data are the mean ± SEM of triplicate wells, with four to six mice in each group. **P < 0.01, WT *vs.* Mysm1^−/−^. (**f**) Mice were immunized with NP-KLH (100 μg in alum) and boosted with NP-KLH (50 μg in PBS) 42 days later. Serum was collected before boost immunization and 5 days after boost and analyzed by ELISA with NP26-BSA-coated plates for detecting NP-specific IgG1, IgG2a, IgG2b, and IgG3 antibodies. (**g**,**h**) Frequencies of total (NP26) and high-affinity (NP4) NP-specific-IgG1 secreting cells (ASCs) in spleen 42 days after primary immunization (**g**) and day 5 after boost (**h**). Data are the mean ± SEM of triplicate wells, with four to six mice in each group, and representative of two experiments. **p < 0.01, Mysm1^−/−^ *vs.* WT.

To further investigate antibody responses in Mysm1^−/−^ mice, we used multiple color cytometry of antigen binding and cell surface phenotype to quantify NP^+^ B cells and plasma cells in Mysm1^−/−^ mice immunized with NP-KLH. Figure 2a,b shows an increase in the frequencies of NP^+^B220^+^ B cells and NP^+^CD138^+^ plasma cells 14 days after primary intraperitoneal immunization with NP-KLH (100 μg) precipitated in alum. Isotype-switched B cells (IgM^−^IgD^−^Gr^−^1^−^CD138^−^B220^+^) were analyzed for NP^+^IgG1^+^ status with NP^+^IgG1^+^ cells being subdivided into GC (CD38^−^) and memory (CD38^+^) B cells. Figure 2c shows an increase in the CD38^+^NP^+^IgG1^+^ memory B cell population in the immunized Mysm1^−/−^ mice. Frozen spleen sections from WT and Mysm1^−/−^ mice 14 days after immunization with NP-KLH were stained with antibodies to B220 to identify follicles (red) and GL7 for germinal centers (green). Figure 2d shows a defective structural formation in germinal centers of immunized Mysm1^−/−^ mice, although GL7^+^ cells were observed in immunized Mysm1^−/−^ mice. We further used ELISPOT assays to examine the frequencies of total and high-affinity anti-NP IgG secreting cells in the spleen and bone marrow of immunized WT and Mysm1^−/−^ mice. Figure 2e shows an increase in frequencies of both total and high-affinity anti-NP IgG-secreting cells in immunized Mysm1^−/−^ mice. Higher frequencies of long-term NP-specific IgG-secreting cells were detected and robust recall responses against NL-KLH were elicited in Mysm1^−/−^ mice by a second immunization with NP-KLH (Fig. 2f–h). Thus, we unexpectedly found that primary and recall TD antibody responses are maintained or enhanced in Mysm1^−/−^ mice despite the severe defect in FO B cell development.

### MYSM1 intrinsically represses plasma cell differentiation

**Figure 3:**
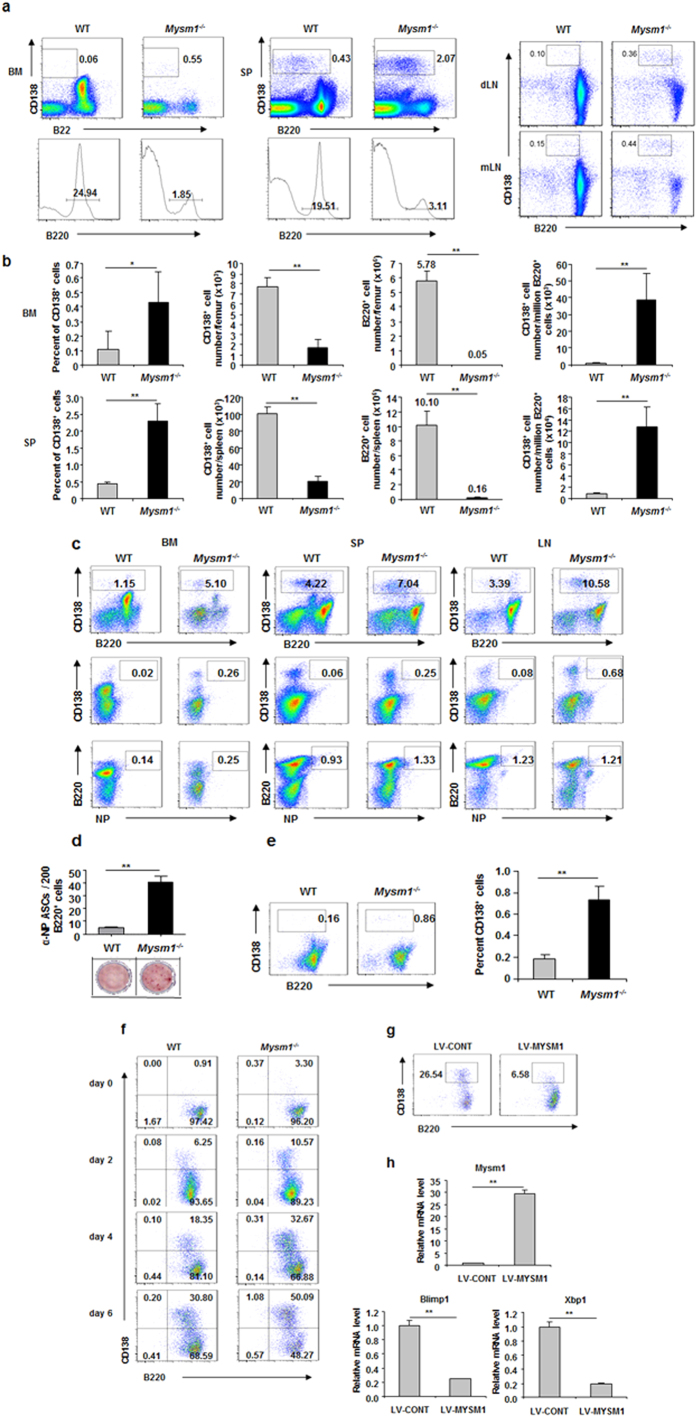
MYSM1 intrinsically represses plasma cell differentiation. (**a**) Representative flow cytometric analysis of CD138^+^ plasma cells in the bone marrow (BM), spleen (SP), draining lymph node (dLN), and mesenteric lymph node (mLN) of naive WT and Mysm1^−/−^ mice. Numbers are the percentage of events within the indicated gates. (**b**) Graphs show the percent of CD138^+^ plasma cells, total number of CD138^+^ cells, total number of B220^+^ cells, and CD138^+^ cells per million B cells in bone marrow (top) or spleen (bottom) of naïve WT and Mysm1^−/−^ mice (n = 5–8 per group). **P < 0.01, WT *vs.* Mysm1^−/−^. (**c**) Flow cytometric analysis of indicated surface markers in the bone marrow, spleen, and lymph node of WT and Mysm1^−/−^ mice 14 days after NP-KLH immunization. (**d**) Splenic B cells from WT and Mysm1^−/−^ mice immunized with NL-KLH were cultured in 96-well plate. Spot numbers (**top**) and representative spots (**bottom**) in the indicated cultures from triplicate wells ± SEM are shown from one of two independent experiments. **P < 0.01, WT *vs.* Mysm1^−/−^. (**e**) Enhanced spontaneous differentiation of plasma cells from naïve splenic Mysm1^−/−^ B cells *in vitro*. Splenic B220^+^ B cells from WT and Mysm1^−/−^ mice were cultured with IL-4 (10 ng/ml) for 4 days. Representative flow cytometric analysis is shown (n = 5–8 per group). **P < 0.01, WT *vs.* Mysm1^−/−^. (**f**) Enhanced plasma cell differentiation of Mysm1^−/−^ B cells after LPS stimulation *in vitro*. Splenic B220^+^ cells from WT and Mysm1^−/−^ mice were cultured with LPS (20 μg/ml) and were collected at indicated days for flow cytometric analysis. (**g**,**h**) MYSM1 rescue assays. Splenic B220^+^ cells from Mysm1^−/−^ mice were transduced with a recombinant lentiviral vector LV-MYSM1 or control vector LV-CONT. The transduced cells were subjected to flow cytometric analysis with indicated antibodies after LPS (20 μg/ml) stimulation *in vitro* (**g**). Quantitative RT-PCR analysis of MYSM1, Blimp1, and Xbp1 mRNA levels in Mysm1^−/−^ splenic B220^+^ cells transduced with LV-CONT or LV-MYSM1 (**h)**. Data were normalized to Hprt and are presented as relative to that of control LV-CONT sample, set as 1, from one of two independent experiments. **p < 0.01, LV-CONT *vs.* LV-MYSM1.

When examining frequencies of plasma cells, we observed higher frequencies of CD138^+^B220^−^ plasma cells in the spleens of naïve Mysm1^−/−^ mice, although the total plasma cell and B cell numbers were drastically lower (Fig. 3a,b). Consistently, we also observed higher frequencies of CD138^+^B220^−^ plasma cells in the BM and lymph nodes (LN) of naïve Mysm1^−/−^ mice. These data suggest an enhanced spontaneous differentiation of B cells into plasma cells in Mysm1^−/−^ mice. To investigate the possible role of MYSM1 in plasma cell differentiation, we used flow cytometry to quantify plasma cells and antigen-specific NP^+^ B cells and plasma cells in Mysm1^−/−^ mice after NP-KLH immunization. Drastic increases in both CD138^+^ plasma cells and antigen-specific NP^+^CD138^+^ plasma cells were observed in various tissues of immunized Mysm1^−/−^ mice (Fig. 3c). Moreover, ELISPOT assays showed higher frequencies of NP-specific, class switched IgG-producing cells (Fig. 3d). Thus, these data indicate that MYSM1 plays a repressive role in plasma cell differentiation.

To investigate whether MYSM1 plays an intrinsic role in plasma cell differentiation, we isolated naïve B220^+^CD138^−^ B cells from WT and Mysm1^−/−^ mice and then cultured the B cells in the presence of IL-4 *in vitro* without additional stimulation. Figure 3e shows an increase in the frequency of CD138^+^ plasma cells in *in vitro* Mysm1^−/−^ B cell cultures, indicating an enhanced spontaneous plasma cell differentiation of Mysm1^−/−^ B cells. When stimulated with LPS to promote plasma cell differentiation, Mysm1^−/−^ B cells were more efficiently differentiated into CD138^+^ plasma cells *in vitro*, as demonstrated by flow cytometry assays (Fig. 3f).

Furthermore, we performed an MYSM1 rescue assay to determine the role of MYSM1 in plasma cell differentiation. Splenic B220^+^ cells from naïve Mysm1^−/−^ mice were transduced with a recombinant lentiviral vector LV-MYSM1 or control vector LV-CONT. The transduced cells were examined by flow cytometry analysis after LPS stimulation. Figure 3g shows that forced expression of MYSM1, but not control, rescued the enhanced plasma cell differentiation of Mysm1^−/−^ B cells. A reduction in Blimp1 and Xbp1 expression was observed with forced expression of MYSM1 in Mysm1^−/−^ B cells (Fig. 3h). Taken together, these data demonstrate that MYSM1 is an intrinsic repressor of plasma cell differentiation.

### MYSM1 intrinsically represses Ig production by plasma cells

**Figure 4:**
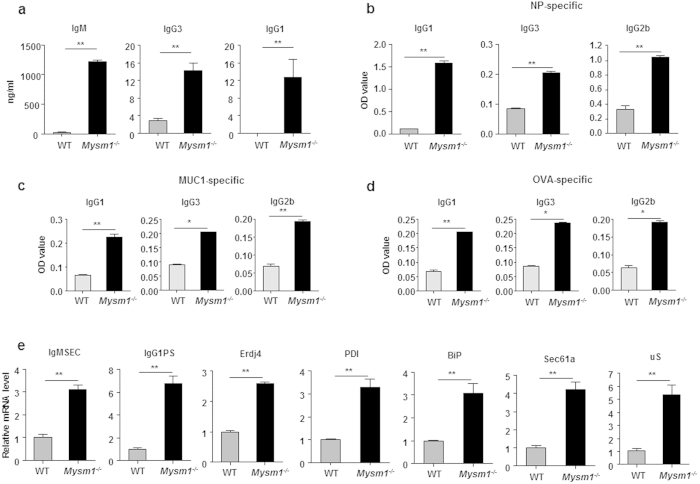
MYSM1 intrinsically represses Ig production by plasma cells. (**a**) Plasma cells (CD138^+^) from naïve WT and Mysm1^−/−^ mice were cultured at 10,000 cells/well in triplicate. Supernatants were collected at 36 h and were assayed for total IgM, IgG3, and IgG1 secretion by ELISA. (**b**–**d**) Supernatant ELISA analysis of NP-specific (**b**), MUC1-specific (**c**), or OVA-specific (**d**) IgG1, IgG3, and IgG2b secretion in sorted CD138^+^ cells from WT and Mysm1^−/−^ mice that were immunized with the indicated antigens in alum. CD138^+^ cells were seeded at 10,000 cells/well in triplicate and the supernatants were harvested after 36 h of culture. (**e**) Quantitative RT-PCR analysis of mRNA levels of indicated genes in sorted CD138^+^ cells from WT and Mysm1^−/−^ mice. Data were normalized to Hprt and is presented as relative to that of WT sample, set as 1, from one of two independent experiments. **p < 0.01, Mysm1^−/−^ *vs.* WT.

Even with enhanced plasma cell differentiation, the total numbers of plasma cells in Mysm1^−/−^ mice were still significantly lower than those in WT littermates (Fig. 3b). Accordingly, we investigated whether MYSM1 controls Ig production by plasma cells, in addition to its role in repressing plasma cell differentiation. We isolated CD138^+^B220^−^ plasma cells from naïve WT and Mysm1^−/−^ mice and seeded an equal number of plasma cells on 96-well plates. The supernatants were harvested for ELISA assays. Figure 4a shows a drastic increase in the production of total IgM, IgG1, and IgG3 production by Mysm1^−/−^ CD138^+^ plasma cells, indicating an enhanced ability of Mysm1^−/−^ plasma cells to produce Ig. To further test this possibility, we isolated CD138^+^ plasma cells from WT and Mysm1^−/−^ mice that were immunized with NP-KLH and seeded an equal number of isolated plasma cells onto plates. In agreement, the production of IgG antibodies against NP, including IgG1, IgG2b, and IgG3 isotypes, by plasma cells from immunized Mysm1^−/−^ mice was also drastically enhanced (Fig. 4b). To further examine the repressive role of MYSM1 in antibody production, Mysm1^−/−^ and WT mice were immunized with OVA, or 20-mer MUC1 peptides emulsified in IFA^25^,^26^. The production of anti-MUC1 IgG1, IgG3, and IgG2b was significantly enhanced by sorted CD138^+^ Mysm1^−/−^ plasma cells from the immunized mice (Fig. 4c). Enhanced levels of anti-OVA IgG1, IgG3, and IgG2b were also produced by CD138^+^ Mysm1^−/−^ plasma cells from the immunized mice (Fig. 4d). In addition, the expression of the representative genes that are important for Ig production was all significantly enhanced in CD138^+^ Mysm1^−/−^ plasma cells (Fig. 4e). Thus, these data demonstrate that MYSM1 represses plasma cell differentiation, as well as Ig production by plasma cells.

### Reduced expression of Pax5 and Bach2 and increased expression of Blimp1 and Xbp1 in Mysm1^−/−^ B cells

**Figure 5:**
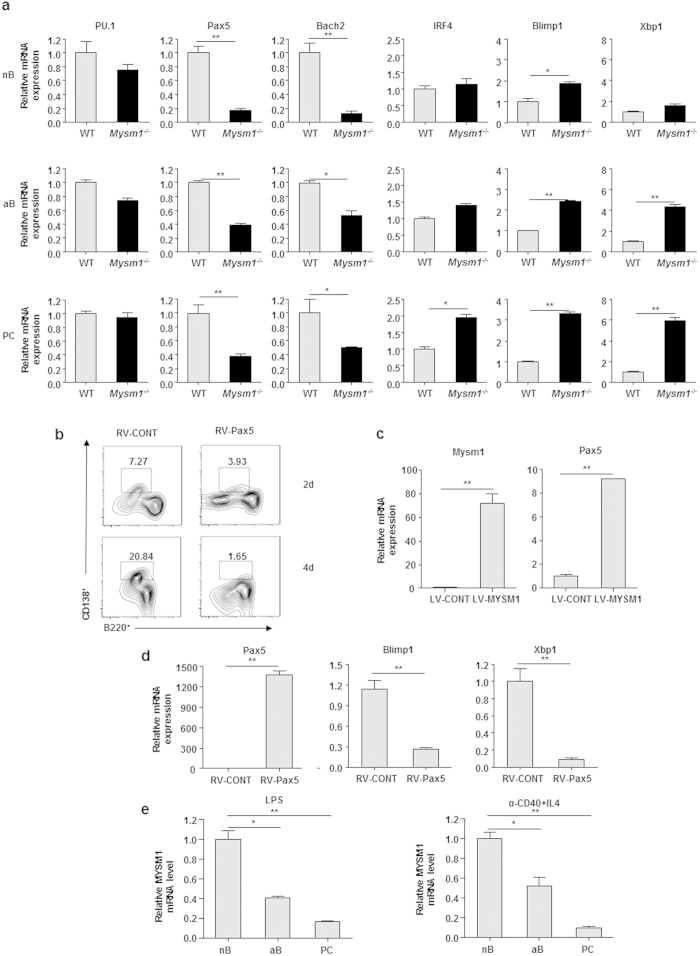
Reduced expression of Pax5 and Bach2 and increased expression of Blimp1 and Xbp1 in Mysm1^−/−^ B cells. (**a**) qRT-PCR analysis of mRNA levels of representative genes in sorted WT and Mysm1^−/−^ cells. Naive splenic B220^+^ cells (nB) from WT and Mysm1^−/−^ mice were stimulated with LPS (20 μg/ml) *in vitro* and, 5 days later, LPS-activated B cells (aB, B220^+^CD138^−^) and plasma cells (PC, CD138^+^ B220^+/−^) were sorted by FACS. Relative mRNA levels were normalized by Hprt mRNA expression and calculated relative to the mRNA expression seen in the WT cells, set as 1. Data are representative of three independent experiments. **P < 0.01, *P < 0.05, WT *vs.* Mysm1^−/−^. (**b**) Forced expression of Pax5 reversed the enhanced plasma cell differentiation from Mysm1^−/−^ B cells *in vitro*. Splenic B220^+^ cells from Mysm1^−/−^ mice were transduced with a recombinant retroviral vector that expresses mouse Pax5, or control vector (RV-CONT). The transduced cells were stimulated with LPS (20 μg/ml), and, 2 and 4 days later, flow cytometric analysis was performed with indicated antibodies. (**c**) Enhanced expression of endogenous Pax5 by forced expression of MYSM1. Mysm1^−/−^ B cells were transduced with LV-MYSM1 or control vector, and 24 hr later mRNA were isolated for qRT-PCR. Relative mRNA levels were normalized by Hprt mRNA expression and calculated relative to the mRNA expression seen in the cells transduced with control vector (LV-CONT), set as 1. Data are representative of two independent experiments. **P < 0.01, LV-CONT *vs.* LV-MYSM1. (**d**) Quantitative RT-PCR of representative genes in transduced Mysm1^−/−^ B cells. Splenic Mysm1^−/−^ B220^+^ cells were transduced with indicated recombinant retroviral vectors RV-Pax5 and stimulated with LPS (20 μg/ml). After 4 d stimulation, cells were collected for qRT-PCR. Relative mRNA levels were normalized by Hprt mRNA expression and calculated relative to the mRNA expression seen in the cells transduced with control vector (RV-CONT), set as 1. Data are representative of two independent experiments. **P < 0.01, RV-CONT *vs.* RV-Pax5. (**e**) MYSM1 mRNA levels in indicated sorted WT naïve B cells (nB), LPS-activated B cells (aB), and plasma cells (PC) from one of three independent experiments. **P < 0.01, nB *vs.* PC *P < 0.05, nB *vs.* aB.

To investigate the mechanism by which MYSM1 represses plasma cell generation and Ig production, we first examined the expression of a set of transcriptional factors that are critical for plasma cell generation and Ig production^27^,^28^,^29^ by qRT-PCR assays. Naïve B cells, activated B cells and plasma cells were FACS sorted. Figure 5a shows that mRNA levels of Pax5 and Bach2 were significantly reduced, while Blimp1 and Xbp1 mRNA levels were significantly elevated in naïve and activated Mysm1^−/−^ B cells. In agreement, Blimp1 and Xbp1 mRNA levels were drastically elevated and Pax5 and Bach2 mRNA levels were significantly reduced in CD138^+^ Mysm1^−/−^ plasma cells derived from naïve B cells after LPS stimulation. We noticed that there was little difference in the expression of Bcl6, a transcriptional repressor of Blimp1 and Xbp1, in Mysm1^−/−^ B cells and plasma cells compared to that in WT cells (data not shown). To confirm this observation, we sorted NP^+^B220^+^CD138^−^ B cells and NP^+^CD138^+^B220^−^ plasma cells from Mysm1^−/−^ and WT mice immunized with NP-KLH for qRT-PCR assays and also observed a significant reduction in Pax5 and Bach2 expression and an increase in Blimp1 and Xbp1 mRNA in the Mysm1^−/−^ B cells and plasma cells (data not shown).

In an earlier study, we observed the drastic reduction in Pax5 and EBF1 expression in B cell progenitors of Mysm1^−/−^ mice^21^. To examine the possibility that the reduced expression of Pax5 in Mysm1^−/−^ B cells contributes to the enhanced plasma cell differentiation, we transduced Mysm1^−/−^ B cells with retroviral vectors that express Pax5 (RV-Pax5) or control RV-CONT, and then observed plasma cell differentiation. Figure 5b shows that forced expression of Pax5 rescued the phenotype of enhanced plasma cell differentiation from Mysm1^−/−^ B cells. To further test the role of MYSM1 in regulating Pax5, Bach2, Blimp1, and Xbp1 expression, we transduced Mysm1^−/−^ B cells with LV-MYSM1 or LV-CONT control and examined mRNA levels of these genes in transduced Mysm1^−/−^ B cells at different days after LPS stimulation. Figure 3h shows a reduction in Blimp1 and Xbp1 expression by forced expression of MYSM1 in Mysm1^−/−^ B cells. In contrast, the expression of Pax5 was significantly enhanced in MYSM1-transduced Mysm1^−/−^ B cells (Fig. 5c). To investigate whether enhanced expression of Pax5 represses the expression of Blimp1 and Xbp1, we transduced Mysm1^−/−^ B cells with RV-Pax5 and examined Blimp1 and Xbp1 mRNA levels in transduced Mysm1^−/−^ B cells after LPS stimulation. Figure 5d shows a reduction in Blimp1 and Xbp1 expression in Mysm1^−/−^ B cells by forced expression of Pax5. Moreover, we examined the expression level of MYSM1 during plasma cell differentiation. Figure 5e shows that MYSM1 was expressed in naïve WT B cells, but its expression was significantly downregulated in activated B cells and differentiated plasma cells. Taken together, these data suggest that MYSM1 is required for the transcription of Pax5, the transcriptional repressors of Blimp1 and Xbp1, and subsequently the repression of plasma cell differentiation and Ig production.

### MYSM1 occupies the Pax5 loci in resting B cells, but not in activated B cells and plasma cells

**Figure 6:**
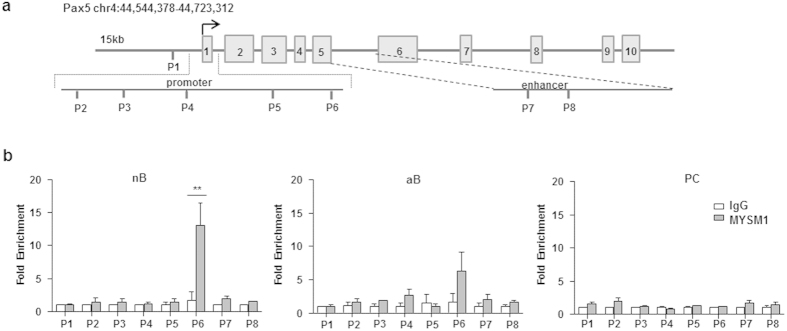
MYSM1 occupies the Pax5 loci in naive B cells. (**a**) Schematic diagram of Pax5 gene and its promoter and enhancer region illustrating the positions of the primer pairs used for ChIP assays. (**b**) ChIP assays of naïve splenic WT B220^+^ cells (nB, left), LPS-activated WT B220^+^CD138^−^ cells (aB, middle), and WT CD138^+^B220^−^ plasma cells (PC, right) using a MYSM1 antibody or control IgG probing for the Pax5 locus. Quantitative PCR was used to analyze the enrichment and the fold enrichments are represented from one of three independent experiments. **p < 0.01, IgG vs. MYSM1.

Further, we tested the possibility that MYSM1 may directly activate the transcription of Pax5 in mature B cells as well. We first examined the association of MYSM1 with the Pax5 locus by ChIP assays with a panel of primer pairs corresponding to the promoter and enhancer regions of the Pax5 locus. Figure 6a,b show that MYSM1 was associated with the promoter region of the Pax5 locus in naïve B cells, suggesting a direct role of MYSM1 in Pax5 transcription. When comparing the occupancy of MYSM1 at the Pax5 locus during plasma cell differentiation, we unexpectedly found that the occupancy of MYSM1 at the Pax5 locus was drastically reduced in activated B cells and plasma cells (Fig. 6b). Together these unexpected observations imply that MYSM1 occupies the Pax5 loci in naïve B cells, but dissociates from the target loci during B cell activation and plasma cell differentiation either due to the reduced MYSM1 expression, as observed in Fig. 5e, or other unknown reasons.

### MYSM1 interacts with the transcription factor PU.1 for its recruitment to the Pax5 locus

**Figure 7:**
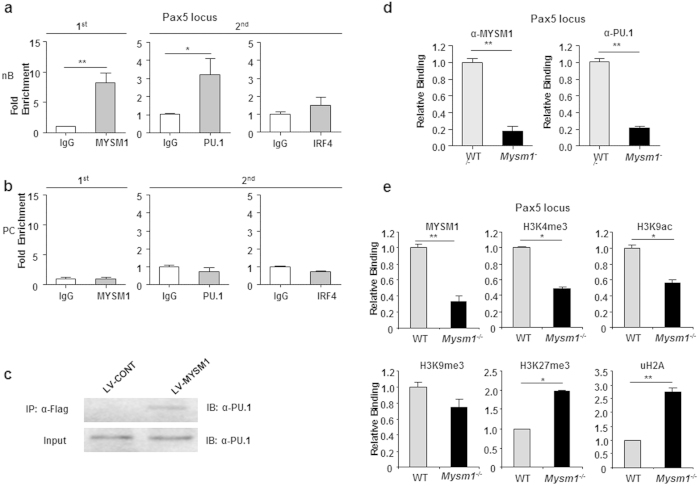
MYSM1 interacts with the transcription factor PU.1 for recruitment to the Pax5 locus. (**a**,**b**) Sequential two-step ChIP assays of naïve splenic WT B220^+^ cells (**a**) and WT CD138^+^B220^−^ plasma cells (**b**) were performed, showing the recruitment of the endogenous PU.1 and MYSM1 to the Pax5 promoter in naïve WT B cells, but not in WT plasma cells, from one of two independent experiments. The relative binding was defined by determining the immunoprecipitation level (ratio of the amount of immunoprecipitated DNA to that of the input sample) and then comparing to corresponding first ChIP or second ChIP control IgG immunoprecipitation level, which was set as 1.0. **P < 0.01, IgG *vs.* MYSM1, *P < 0.05, IgG *vs.* PU.1. (**c**) Co-immunoprecipitation of PU.1 and MYSM1. Cell lysates from splenic WT B cells transduced with lentivirus containing Mysm1 vector (LV-MYSM1) or control flag vector (LV-Flag) were immunoprecipitated with anti-Flag antibody, then probed with a PU.1 antibody. Five percent of the cell lysate input was loaded. (**d**) Drastic reduction of PU.1 occupancy at the Pax5 promotion region of naïve Mysm1^−/−^ B cells, compared to that of naïve WT B cells. ChIP data are presented from one of two independent experiments. **P < 0.01, WT *vs.* Mysm1^−/−^. (**e**) Altered histone modifications at the Pax5 locus in splenic Mysm1^−/−^ B cells. ChIP analysis of naïve splenic WT or Mysm1^−/−^ B cells. The DNA precipitated with the indicated antibodies was analyzed by quantitative PCR with primers amplifying the Pax5 promoter region and normalized with input DNA before being compared to WT (set as 1). Data are presented from one of two independent experiments. **P < 0.01, *P < 0.05, WT *vs.* Mysm1^−/−^.

MYSM1 was found to regulate target gene transcription by directing histone H2A deubiquitination and additional histone modifications, and transcription factor recruitment to target loci^21^. Several transcription factors, such as PU.1, EBF1, IRF4, and NF-kB, were found to activate Pax5 transcription in B cells^30^. To investigate whether MYSM1 interacts with the transcriptional factors of Pax5 at the Pax5 locus, we performed a sequential two-step ChIP assay with the first anti-MYSM1 ChIP, followed by the second ChIP with one of the antibodies against these known transcription activators for Pax5 transcription^21^. Through the sequential two-step ChIP assays, we found that MYSM1 was associated with PU.1 at the Pax5 locus in naïve WT B cells (Fig. 7a). However, the association of MYSM1 with IRF4 (Fig. 6b), Stat3/5, NF-kB, and AP-1 at the Pax5 locus in naïve WT B cells was not positively identified in our assays. Consistent with earlier observations (Fig. 6b), the association of MYSM1 and PU.1 with the Pax5 locus was not detected in WT plasma cells. We then used co-immunoprecipitation assays to confirm the association. MYSM1 protein was found to co-precipitate with endogenous PU.1 proteins in naïve WT B cells (Fig. 7c). Furthermore, we tested whether MYSM1 is required for the recruitment of PU.1 to the Pax5 locus in naïve B cells by ChIP assays of naïve WT and Mysm1^−/−^ B cells. Figure 7d shows that the transcription factor PU.1 was associated with the Pax5 locus in WT B cells, but this association was not detected in Mysm1^−/−^ B cells, indicating a critical role of MYSM1 in the recruitment of PU.1 to the Pax5 locus in naïve B cells. Moreover, we used ChIP assays with antibodies against various histone markers to investigate whether histone modifications at the target Pax5 locus were altered in Mysm1^−/−^ B cells. We observed an increase in ubH2A levels at the Pax5 promoter region of Mysm1^−/−^ B cells (Fig. 7e). We also examined the levels of representative histone modifications that are known to be associated with transcriptional activation (H3K4me3 and H3K9ac) or repression (H3K27me3 and H3K9me3), and saw an increase in the levels of repressive marks (H3K27me3) and a decrease in the levels of active marks (H3K4me3 and H3K9ac) at the Pax5 locus of the Mysm1^−/−^ B cells as compared to WT B cells (Fig. 7e). Together, these data indicate that MYSM1 likely plays a critical role in regulating histone modifications at the target Pax5 locus and that MYSM1 is required for the recruitment of the transcription factor PU.1 to the Pax5 locus in naïve B cells.

## Discussion

In this study, we found that mice deficient in the histone H2A deubiquitinase MYSM1, despite their severe defect in FO B cell development, exhibit an enhanced antibody response against both T cell-dependent and –independent antigens. We demonstrated that MYSM1 intrinsically represses plasma cell differentiation and antibody production. Mechanistic studies revealed that MYSM1 is a transcriptional activator of Pax5, the repressors of plasma cell differentiation, by facilitating key transcriptional factor recruitment and coordinating histone modifications at the Pax5 locus in B cells. Hence, this study uncovers a critical role for MYSM1 in epigenetically repressing plasma cell differentiation and antibody production, in addition to its opposing, active role in B cell development.

Vaccines are aimed to induce high levels of protective antibodies against pathogens, while many autoimmune and allergic diseases result from dysregulated antibody responses. Development of antibody-secreting plasma cells from B cells is the critical event in driving the humoral immune response after B cells are activated. Plasma cells that secrete antibodies with high affinity for TD antigen, as well as memory B cells are mainly generated from FO B cells^22^,^23^,^24^,^31^. A complex transcriptional program that includes a set of interacting positive and negative regulators dictates plasma cell differentiation from B cells and Ig production^27^,^28^,^29^. Although the expression of the transcription factors Blimp1, IRF4, and Xbp1 is critical for promoting plasma cell differentiation and Ig production, the initiating event of plasma cell differentiation is the downregulation of Pax5, Bach2, and other transcriptional repressors of Blimp1 and Xbp1, which precedes the upregulation of Blimp1 and Xbp1 transcription^27^,^28^,^29^. Pax5 is essential for B cell commitment and development^32^, as well as directly repressing Prdm1 that encodes Blimp1 in B cells^33^. Importantly, inactivation of Pax5 in DT40 B cells led to spontaneous differentiation to plasma cells^34^. A rapid decrease in Pax5 and Bach2 levels before induction of Blimp1 mRNA was found in human tonsillar centrocytes that were stimulated to drive plasmacytic differentiation^35^. In agreement, Kallies *et al.* found that the expression of Pax5 was downregulated prior to the upregulation of Blimp1 expression in pre-plasmablasts^36^. Collectively, these data indicate that removal of transcriptional repressors such as Pax5 initiates plasma cell differentiation, which cannot be explained by Blimp1-mediated transcription repression of these repressors. To date, the mechanism for downregulating Pax5 expression after B cells are activated is still unknown. In this study, we unexpectedly found that primary and recall TD antibody responses were enhanced in Mysm1^−/−^ mice, despite their severe defect in B cell development, that was reported in our recent study^21^. We further found that mRNA levels of Pax5 and Bach2, the transcriptional repressors of Blimp1 and Xbp1, were significantly reduced in Mysm1-deficient B cells, although the Bcl6 mRNA was not substantially altered. In contrast, there was a drastic increase in Blimp1 and Xbp1 mRNA levels in activated MYSM1-deficient B cells. The reduced Pax5 and Bach2 expression likely contributes to the enhanced expression of Blimp1 and Xbp1 and the subsequent enhancement of plasma cell formation and Ig production in MYSM1-deficient mice. Mechanistic studies revealed that MYSM1 activates Pax5 transcription by coordinating histone modifications and directing the recruitment of the transcription activators such as PU.1 to the Pax5 locus in B cells. Importantly, the occupancy of MYSM1 and its interacting transcription factor PU.1 at the target Pax5 locus is greatly reduced during plasma cell differentiation following B cell activation. We also revealed that Mysm1^−/−^ B cells were prone to spontaneously differentiate into plasma cells and were more efficiently differentiated into plasma cells upon immunization or *in vitro* stimulation. Moreover, we observed that MYSM1 expression was significantly down regulated after B cells were activated and remained at low levels in activated B and plasma cells. These data indicate a critical role of MYSM1 in maintaining the B cell program likely by activating Pax5 transcription in naïve stage. These data further indicate that MYSM1 is a key controller of the initiation of plasma cell differentiation by switching off the transcription of Pax5 after B cells are activated.

High-affinity antibody-secreting plasma cells are usually generated in a GC-dependent follicular reaction^22^,^23^,^24^,^37^. In GCs, antigen-specific B cells undergo extensive proliferation and somatic hypermutation and differentiate into either plasma cells or memory cells. Although anatomic GC was not clearly identified in the spleen and lymph nodes of immunized Mysm1-deficient mice, GC positive B cells were detected by flow cytometry. However, anatomic GCs are not always required for affinity maturation because mice deficient in GCs, such as LT-α–deficient, Lyn-deficient, and Cr2-deficient mice, exhibit measurable affinity maturation after immunization^38^,^39^,^40^. Moreover, in SWAP-70-deficient mice, plasma cell and high-affinity Ab formation can occur at even higher levels when GC formation is severely impaired^41^. Interestingly, Rag1/2 mutations in mice, related to human Omenn syndrome, exhibited a severe B cell developmental block and a normal or enlarged compartment of Ig-producing plasma cells^42^,^43^, which somewhat resembles the B cell phenotype of Mysm1^−/−^ mice described in this study. However, different from Mysm1^−/−^ mice that are able to produce high affinity, class-switched antibodies, TD antibody responses are severely defective in Rag1/2 mutant mice. Nevertheless, this present study raises many interesting questions. For examples, it is not clear how Mysm1^−/−^ B cells achieve efficient proliferation and somatic hypermutations without anatomic GC, while they rapidly differentiate into high affinity antibody-secreting plasma cells. It is also unknown how MYSM1 expression is rapidly downregulated after B cells are activated. Moreover, given that frequencies of NP-specific B cells are also increased in immunized Mysm1^−/−^ mice, MYSM1 likely regulates other genes involved in B cell activation and proliferation. These interesting questions will be investigated in future studies.

## Methods

### Mice and immunization

MYSM1 KO-first (Mysm1^−/−^) mice, in which the MYSM1 mRNA transcript is prematurely truncated with an inserted efficient polyadenylation termination signal, and the floxed MYSM1 exon 3 can be further deleted by crossing with Cre transgenic mice, were generated and maintained, as described in our previous publication^21^. MYSM1 KO-first mice were further crossed with MMTV-Cre mice or Tek-Cre mice to delete the floxed Mysm1 gene^21^. All mice were bred in a pathogen-free barrier facility and experiments were approved and performed in accordance with the University of Southern California Institutional Animal Care and Use Committee. Mysm1^−/−^ mice and littermate control mice, 7–10 weeks old, were immunized intraperitoneally (IP) with either (4-hydroxy-3-nitrophenyl) acetyl (NP)-Ficoll (50 μg of) in 0.1 ml of PBS or 50–100 μg of NP-keyhole limpet hemocyanin (KLH) or recombinant proteins or peptides precipitated with alum or emulsified in an incomplete Freund’s adjuvant (IFA). For a recall response, mice were immunized with the same antigens at least 6 weeks after the initial immunization.

### Flow cytometric analyses and sorting

Cell preparation and cytometric analysis and sorting were performed as described previously^26^,^44^. Single-cell suspensions of bone marrow (BM), spleen, draining lymph nodes and mesentery lymph nodes were prepared and were first incubated for 20 min at 4 °C with CD16/CD32 Fc-blocking antibody (2.4G2), unless indicated otherwise, in flow cytometry buffer, followed by incubation with a ‘cocktail’ of antibody conjugated to fluorescein isothiocyanate (FITC), phycoerythrin (PE), peridinine chlorophyll protein complex–cyanine 5.5 (PerCP-Cy5.5), phycoerythrin-indotricarbocyanine (PE-Cy7), allophycocyanin (APC), or allophycocyanin-indotricarbocyanine (APC-Cy7). For each staining, at least 1,000,000 events were collected for analysis. The following antibodies from BD Biosciences, eBioscience, BioLegend were used for flow cytometry: CD16/32 (2.4G2), CD38 (90/CD38), B220 (RA3-6B2), IgM (331.12), IgD (11-26C), Gr-1 (RB6-8C5), CD138 (281-2), IgG1 (X56), PNA (FL-1071), CD19 (1D3), CD4 (L3T4), CD8a (53-6.7), TCRβ chain (H57-597), IgE (R35-72), IgA (C10-1), GL-7 (GL-7), Fas (Jo2), NP (Biosearch Technologies INC), CD19 (SJ25C1), IgM (G0-127), IgG (G18-145), IgG1 (G17-1), CD38 (HIT), CD20 (HI), CD138 (MI15) and matched isotype controls. Data were collected on a FACSCanto II (BD) and analyzed with FlowJo software (TreeStar). Cells stained with indicated surface markers were isolated with FACSAria cell sorter.

### ELISA and ELISPOT assays

ELISA was used to quantify IgM and IgG levels in cell culture supernatants and levels of IgM, IgG1, IgG2a, IgG2b, IgG3, and IgA in mouse serum as described previously^25^,^26^. To measure the relative amounts of NP-specific serum antibodies in mice immunized with NP-KLH or NP-ficoll, plates were coated with 25 μg/ml NP(25)-BSA or NP(4)-BSA (Biosearch Technology) or MUC1 peptides or OVA proteins (Sigma) overnight at 4 °C. To standardize and quantify relative amounts of NP-specific IgG responses, all experimental samples were compared with a standardized dilution of pooled serum obtained from immunized WT or Mysm1^−/−^ mice. ELISPOT for detection of NP-specific Ig was performed. 96-well multiscreen membrane filtration plates (Millipore) were coated with 25 μg/ml NP(25)-BSA, NP(4)-BSA, MUC1 peptides or OVA proteins overnight at 4 °C. The wells were washed and cells were seeded into each well and incubated for >18 h at 37 °C in 5% CO2. The wells were washed before the addition of horseradish peroxidase goat anti–mouse IgM or IgG (Southern Biotech) for 4 h and developed using 3-amino-9-ethylcarbazole (Sigma-Aldrich). Wells in triplicate were then scored.

### B cell culture

Splenic B cells were enriched by positive selection of B220-expressing cells with CD45R microbeads (Miltenyi Biotech) according to the manufacturer’s instructions. B cell samples were routinely enriched to over 95% B220^+^ cells, as assessed by flow cytometry. Purified B cells were cultured at 1 × 10^5^/ml in medium (RPMI 1640, 10% FBS (Gibco), 1% nonessential amino acids (Invitrogen), 1% oxaloacetate-pyruvate-insulin (OPI) (Invitrogen), 100 U/ml gentamicin, and 50 μM 2-mercaptoethanol) supplemented with optimal concentrations of anti-CD40 antibody (1C10, 10 μg/ml, 3/23; BD Bioscience), LPS (20 μg/ml, Sigma-Aldrich), F(ab’)2 goat anti–mouse IgM (115-006-020; Jackson ImmunoResearch), IL4 (500 U/ml, R&D Systems) or IL5 (2 ng/ml, R&D Systems).

### Lentivirus and retrovirus production and transduction

Recombinant lentiviral vectors were produced as described in our previous publications^45^,^46^,^47^. Lentivirus supernatants were prepared by transient cotransfection of 293T cells with package plasmids VSVg, Rev, Gag/Pol and lentiviral constructs encoding MYSM1-eGFP (LV-MYSM1) or eGFP alone (LV-CONT). Viral supernatants were collected after 60 to 72 hours. Retroviruses were produced as described previously^48^,^49^. Briefly, 293T cells were transfected with plasmids that encode viral proteins (pCL-10A1) and a specific gene expression vector (MSCV) encoding Pax5 followed by an IRES-GFP cassette^48^. Empty MSCV vector expressing GFP alone acted as a control. Viral supernatants were harvested after 48–72 hours. For transduction, splenic B220^+^ cells were cultured in RPMI1640 supplemented with 10% fetal bovine serum (FBS), 100 U/ml penicillin, 100 μg/ml streptomycin, 2 mM L-glutamine, and 50 μM β-mercaptoethanol with or without 20 ug/ml LPS. Lentiviral or retroviral supernatants were applied to culture dishes pretreated with RetroNectin (TaKaRa) and centrifuged at 3,000 rpm for 90 minutes and then incubated at 37 °C in the presence of polybrene (4 μg/ml) for an additional 6 hours. Cells were then washed and resuspended in fresh media.

### Semiquantitative and quantitative RT-PCR

Semiquantitative and quantitative RT-PCR were performed as described previously^21^. Total RNA from isolated cells was purified with RNeasy Microkit (Qiagen) according to the manufacturer’s instructions. The SuperScript III First-Strand Synthesis kit (Invitrogen) was used for reverse transcription. Serially diluted cDNA was used for semiquantitative PCR analysis. A SYBR Green PCR kit (BIO-RAD) was used for quantitative real-time PCR and results were quantified with an ICycler IQ (BIO-RAD). Sequences of primer pairs are available upon request.

### Chromatin Immunoprecipitation

Chromatin was immunoprecipitated according to the manufacturer’s instruction (#9002, Cell Signaling)^21^,^44^. Briefly, sorted cells were crosslinked with 1% (vol/vol) formaldehyde at room temperature for 10 min, and incubated with glycine for 5 min at room temperature. Cells were then sequentially washed in ice-cold buffer A and buffer B, followed by digesting with MNase. Nuclear pellet was suspended in ChIP buffer, sheared by sonication with an average size of sheared fragments of about 300 base pairs (bp) to 800 bp. After centrifugation at 10,000 rpm for 10 minutes, sheared chromatin was diluted in ChIP buffer and precleared by addition of protein A/G plus agarose beads (sc-2003) for 1 h at 4 °C. The beads were discarded and the supernatant was then incubated with one of these antibodies, H3K4me3 (ab-1012), H3K27me3 (ab-6002), H3K9me3 (ab-8898), H3K9ac (ab-4441), uH2A (05–678), PU.1 (T-21, sc-352), Pax5 (c-20, sc-1974), IRF4 (M-17, sc-6059) or control anti-IgG (Cell Signaling), at 4 °C overnight. At the next day, protein A/G plus agarose beads were added and incubated for 2 h at 4 °C. For anti-uH2A, anti-mouse IgMμ (12-488, Millipore) and protein A/G plus agarose beads were added. Beads were harvested by centrifuge and went through 3 low salt washes and one high salt wash. Beads were then eluted with ChIP elution buffer. The elutes and input were then added with proteinase K and RNase A and heated at 65 °C for 2 h to reverse the formaldehyde cross-link. DNA fragments were purified with column. For sequential two-step ChIP experiments^21^, crosslinked chromatin was immunoprecipitated with antibody against MYSM1 or a control IgG (Cell signal, #9002). Precipitated chromatin was then eluted in a solution of 30 mM DTT, 500 mM NaCl, and 0.1% SDS. Eluted chromatin was diluted 5–10 fold with ChIP buffer (Cell signaling, #9002) and then re-immunoprecipitated with one of these antibodies, PU.1 (T-21, sc-352), Pax5 (c-20, sc-1974), IRF4 (M-17, sc-6059) or control IgG. The relative binding was defined by determining the immunoprecipitation level (ratio of the amount of immunoprecipitated DNA to that of the input sample) and then comparing to corresponding 1^st^ ChIP or 2^nd^ ChIP control IgG immunoprecipitation level, which was set as 1.0.

### Western blotting

Western blotting analysis was performed as described^44^. Cell lysates or nuclear and cytoplasmic fractions were produced from mouse tissues, precipitated with an antibody specific for Flag (Sigma) and subjected to SDS-PAGE then western blotting was performed using standard techniques. Membranes were probed with an antibody specific for PU.1 (Santa Cruz).

### Statistics

Groups of three to eight mice were used for statistical analysis. *P* values were calculated with Student’s t-test.

## Additional Information

**How to cite this article**: Jiang, X.-X. *et al.* Epigenetic Regulation of Antibody Responses by the Histone H2A Deubiquitinase MYSM1. *Sci. Rep.* **5**, 13755; doi: 10.1038/srep13755 (2015).
